# Sex-based diagnostic bias in AI symptom checkers: a structured, exploratory audit of three platforms

**DOI:** 10.3389/fdgth.2026.1950777

**Published:** 2026-10-08

**Authors:** Samah Khan, Michael K. Paasche-Orlow

**Affiliations:** 1 Department of Social and Behavioral Sciences, New York University School of Global Public Health, New York, NY, United States; 2 Department of Medicine, Division of General Internal Medicine, Tufts University School of Medicine, Boston, MA, United States; 3 Department of Medicine, Tufts Medical Center/Tufts Medicine, Boston, MA, United States

**Keywords:** algorithmic bias, artificial intelligence, diagnostic errors, health equity, sex bias, symptom checkers

## Abstract

Artificial intelligence symptom checkers are used by hundreds of millions of people annually as first-line triage tools, and their outputs may influence whether and how quickly a person seeks emergency care. We conducted a structured, exploratory audit of three widely used platforms, Ada Health, Isabel Healthcare, and Docus AI, across four standardized clinical vignettes [acute coronary syndrome (ACS) with chest pressure, ACS without chest pressure, pulmonary embolism, and panic attack], entering each vignette for matched male and female synthetic patient profiles with sex as the sole variable altered. We observed sex-based divergence in outputs from all three platforms, appearing as differences in differential diagnosis rank, differences in assigned probability, and differences in generated explanatory or advisory text. The direction of this divergence was not uniform: some findings were unfavorable to the female profile and others were favorable to it. Isabel Healthcare ranked Heart Attack first for the male profile but third for the female profile on the ACS with chest pressure vignette, and ranked Heart Attack seventh rather than fifth for the female profile on the panic attack vignette; on the ACS without chest pressure vignette, the same platform ranked Heart Attack first for the female profile, ahead of the male profile. Docus AI assigned a lower ACS probability to the female profile on the ACS without chest pressure vignette while its own text described this presentation as common in women, and applied a mirrored justification, common in males, to support a higher probability for the male profile given identical symptoms; the platform's closing advisory text also described immediate medical attention as crucial for the male profile and prompt evaluation as merely important for the female profile. Ada Health's explanatory text for the female profile stated that heart attacks are slightly more common in men than women, presented alongside a demoted cardiac ranking. These findings indicate that AI symptom checkers can produce sex-based divergence in their outputs, including an internally inconsistent, self-contradictory justification on one platform, and support the case for equity auditing of such systems, with attention to both numerical outputs and generated explanatory text, before and after deployment.

## Introduction

Artificial intelligence (AI) symptom checkers are increasingly used as first-line triage tools, with some platforms reporting tens of millions of users annually (1). These systems generate differential diagnoses, probability estimates, and triage urgency recommendations based on user-reported symptoms, and these outputs may directly influence care-seeking behavior.

Sex and gender differences in clinical presentation and in the broader practice of medicine are well documented (2, 3), including within the cardiology literature specifically (4–7). Women presenting with acute coronary syndrome (ACS) are less likely to receive timely evaluation, in part because their symptom profiles more frequently include fatigue, jaw pain, nausea, and dyspnea rather than chest pressure (4, 6, 7), and some evidence suggests these presentations are underrecognized in clinical settings (8). Population-level data show that women are more likely than men to present with ACS without chest pressure (4, 6); the individual-level predictive value of chest pressure for ACS, however, does not appear to differ by sex to the same degree (7). If AI triage systems misapply population-level epidemiological patterns to individual clinical encounters, they risk automating and scaling diagnostic inequity across the populations that rely on these tools.

Prior work has identified algorithmic bias in clinical risk scores, pulse oximetry, and imaging AI (9–11). Direct empirical audits of consumer-facing AI symptom checkers for sex-based differential diagnosis divergence are, to our knowledge, lacking. We conducted a structured, exploratory audit to assess whether three widely used AI symptom checker platforms produce different outputs for male and female patient profiles given identical symptom inputs, and to describe the observable patterns through which any such divergence occurred.

## Method

### Platform selection

Three AI symptom checker platforms were selected to represent a range of architectural approaches and intended-user populations: Ada Health (Berlin, Germany), a conversational AI system using dynamic symptom intake and reporting more than 15 million users (1); Isabel Healthcare (Woodbridge, UK), a clinical decision support tool using static free-text symptom entry that is marketed to both patients and clinicians; and Docus AI (Wilmington, DE, USA), a consumer-facing symptom checker generating probability-labeled differentials through a generative, conversational interface. These three platforms span static free-text entry, adaptive conversational intake, and generative language-model output, which we judged to capture much of the architectural diversity present in publicly available symptom checkers. Findings from three platforms cannot be assumed to generalize to the broader category of consumer symptom checkers.

### Vignette design

Four standardized clinical vignettes were developed: (1) ACS with chest pressure (chest pressure radiating down the left arm, nausea, shortness of breath); (2) ACS without chest pressure (fatigue, jaw ache, upper back pain, nausea, shortness of breath, no chest pain); (3) pulmonary embolism (sudden sharp pleuritic chest pain, shortness of breath, tachycardia); and (4) panic attack (palpitations, chest tightness, lightheadedness, tremor, shortness of breath). The two ACS vignettes were constructed, respectively, to reflect a presentation classically associated with male patients and a presentation classically associated with female patients, drawing on the symptom clusters described in the cited cardiology literature (4–7). The pulmonary embolism and panic attack vignettes served as non-cardiac reference scenarios, allowing us to assess whether any observed divergence was specific to cardiac presentations or reflected a broader pattern. These vignettes were constructed by the authors based on the published literature. Symptom text was held verbatim across patient profiles for the two static-entry platforms (Isabel Healthcare, Docus AI); for Ada Health's adaptive conversational intake, the same opening symptom description was entered for both profiles, and any dynamically generated follow-up questions were answered with matched, clinically equivalent responses, as detailed below.

### Data collection protocol

All three platforms were accessed via their publicly available web interfaces between 25 and 26 March 2026. Each vignette and profile combination was entered as a new, freshly cleared browser session with no prior interaction history. Each vignette was entered for a male profile (John, age 40–49, United States) and a female profile (Jane, age 40–49, not pregnant, United States), with sex as the sole variable altered. Across all three platforms and all four vignettes, the male profile was tested immediately before the female profile, with no alternation of order; we report this as a limitation, since we cannot rule out order or session-state effects contributing to some of the divergence observed, particularly on the adaptive and generative platforms.

For Ada Health, which poses dynamic follow-up questions, we answered with matched, clinically equivalent responses across profiles wherever an equivalent question was posed. Any deviations from this intended equivalence, identified on *post hoc* review of the underlying assessment logs, are reported in the Results.

Exact vignette text, session timestamps, and, for Ada Health, the complete follow-up question and answer logs for all 24 platform, vignette, and profile combinations are provided in Supplementary Table S1 and archived with platform screenshots at the Open Science Framework (OSF) repository referenced in the Data Availability Statement.

All three platforms required a structured intake step, separate from the free-text or conversational symptom description, that captured sex assigned at birth and, where applicable, pregnancy status, prior to generating a differential. For Isabel Healthcare, this is directly visible in the exported report, which displays discrete Age, Sex, Pregnancy, and Country fields ahead of the symptom text. For Ada Health and Docus AI, this occurred in a structured intake screen presented before the conversational or chat interface opened; this screen preceded the exported assessment summary and was not itself captured in the archived report, but is reflected in Ada Health's downstream branching (for example, a pregnancy status question was posed to the female profile and no equivalent item appears for the male profile). Because sex was entered as a discrete demographic field on all three platforms rather than inferred from the patient's name, we consider it unlikely that any observed divergence reflects a name-based linguistic association rather than the platforms' handling of an explicitly stated clinical variable.

### Outcome measures

Platform outputs were captured via screenshot and text extraction. Outcomes assessed included: (1) rank of ACS or Heart Attack in the differential diagnosis list; (2) probability label or estimate assigned to ACS; (3) presence of sex-specific and clinically irrelevant diagnoses; (4) content of intake questions posed to male versus female profiles; (5) presence of explicit sex-based framing language in platform-generated explanatory text; and (6) where available, differences in stated urgency or care-seeking recommendations. Outputs were compared qualitatively across paired male and female vignettes for each platform and scenario. This was a single observation pass per platform and vignette; we did not assess variability across repeated sessions.

### Ethical considerations

This study analyzed outputs of publicly accessible AI platforms using synthetic patient profiles entered through each platform's standard web interface. No human participants, patient data, or identifiable health information were involved, and no platform accounts or real personal information were required to complete any of the assessments. We reviewed each platform's publicly posted terms of use prior to testing; none of the three platforms' terms prohibited this kind of manual, non-automated evaluation of publicly available outputs. We recognize that independently auditing and publicly reporting on the outputs of deployed commercial health AI systems raises its own ethical considerations, including potential reputational impact on the audited companies. We consider this outweighed by the public interest in understanding how widely used, consumer-facing health AI systems perform for different users, consistent with established practice in algorithmic bias auditing of other high-stakes commercial systems (9–11). Accordingly, this study was exempt from institutional ethics review.

## Results

Across all three platforms, we observed sex-based divergence in differential diagnosis rank, assigned probability, and generated explanatory or advisory text. Findings are reported by platform below in a consistent order (rank, probability, and text, by vignette) and summarized in Table 1. We report observed outputs here; candidate explanations for these outputs are discussed separately below.

**Table 1:** Summary of observed sex-based findings across AI symptom checker platforms and clinical vignettes.

Platform	Scenario	Male (John)	Female (Jane)	Observed sex-based finding
Isabel Healthcare	ACS with chest pressure	Heart Attack #1	Heart Attack #3; Pelvic Organ Prolapse in differential	Rank difference; sex-irrelevant diagnosis present for female profile only
Isabel Healthcare	ACS without chest pressure	Heart Attack #3 (SCAD #1, Thoracic Aortic Aneurysm #2)	Heart Attack #1	Rank difference, favorable to female profile
Isabel Healthcare	Pulmonary embolism	Pneumothorax #1; PE #2	Pneumothorax #1; PE #2	No difference observed
Isabel Healthcare	Panic attack	Heart Attack #5; Panic Attack #18	Heart Attack #7; Panic Attack #18	Heart Attack rank difference (#5 to #7); Panic Attack rank identical for both
Docus AI	ACS with chest pressure	ACS: High	ACS: High	No difference observed
Docus AI	ACS without chest pressure	ACS: High; text cites atypical presentation especially in males; advisory text describes immediate attention as crucial	ACS: Moderate; text cites atypical presentation especially in women; advisory text describes prompt evaluation as important	Probability difference; mirrored, sex-matched text justifying opposite estimates; urgency-language difference in advisory text
Docus AI	Pulmonary embolism	Pneumothorax #1 (Moderate to High); PE #2 (Moderate)	PE #1 (Moderate to High); Pneumothorax #2 (Moderate)	Rank difference, favorable to female profile
Docus AI	Panic attack	Panic Attack #1 (High); ACS #2 (Moderate); text notes age and gender should be considered for ACS	Panic Attack #1 (High); ACS #3 (Moderate to Low); no equivalent age/gender text generated	ACS rank difference (#2 to #3); sex-framing text present for male profile only
Ada Health	ACS with chest pressure	Heart Attack #1 (5/10); Unstable Angina #2 (8/100)	Heart Attack #1 (4/10); Unstable Angina #2 (1/10)	Probability difference for both primary and secondary diagnosis
Ada Health	ACS without chest pressure	Hyperventilation #1 (2/10); PE #2; Heart Attack #3 (6/100)	Hyperventilation #1 (3/10); PE #2; Acute Resp. Failure #3; Heart Attack #5 (3/100); sex-framing text on 3 of top 5 entries	Rank difference (#3 to #5); probability difference; sex-framing text present for female profile only
Ada Health	Pulmonary embolism	PE #1 (3/10); Collapsed Lung #2 (2/10); Heart Attack #3 (1/10)	Heart Attack #1 (2/10); Collapsed Lung #2 (1/10); PE #3 (1/10)	Rank difference: PE and Heart Attack rank order reversed
Ada Health	Panic attack	Panic Attack #1 (4/10); tense chest muscles entry; no distinct chest pain entry	Panic Attack #1 (4/10); distinct structured chest pain entry generated; no rank or probability difference	No difference in top-ranked output; intake structure differed (see Limitations)

ACS, acute coronary syndrome; PE, pulmonary embolism; SCAD, spontaneous coronary artery dissection. This table reports directly observed outputs; interpretation of which findings may reflect a concerning pattern is reserved for the Discussion.

### Isabel healthcare

On the ACS with chest pressure vignette, Isabel Healthcare ranked Heart Attack first for the male profile (John) and third for the female profile (Jane), behind Bacterial Pneumonia and Angina. Pelvic Organ Prolapse appeared in Jane's differential; no anatomically sex-linked diagnosis appeared in John's.

On the ACS without chest pressure vignette, this pattern reversed: Isabel ranked Heart Attack first for Jane and third for John, behind Spontaneous Coronary Artery Dissection (SCAD) and Thoracic Aortic Aneurysm.

On the pulmonary embolism vignette, no rank difference was observed: both profiles returned Pneumothorax first and Pulmonary Embolism second.

On the panic attack vignette, Heart Attack was ranked fifth for John, behind Wolff-Parkinson-White Syndrome, Atrial Flutter, Atrial Fibrillation, and Ventricular Tachyarrhythmias. For Jane, Heart Attack was ranked seventh, behind these same four conditions plus Supraventricular Tachycardia and SCAD. Panic Attack itself, the presentation the vignette was designed around, was ranked eighteenth of twenty-two possible diagnoses for both John and Jane, an identical position for both profiles.

Isabel Healthcare's interface did not generate explanatory narrative text alongside its ranked differentials on any of the four vignettes; its outputs consist of a ranked list only, and it did not expose a distinct urgency or care-seeking recommendation beyond rank position.

### Docus AI

On the ACS with chest pressure vignette, Docus AI assigned a High probability to ACS for both profiles; we did not observe a probability or ranking difference on this vignette.

On the ACS without chest pressure vignette, Docus AI assigned a High probability to ACS for John and a Moderate probability to ACS for Jane on identical symptoms. The accompanying text for John stated that these symptoms can present with “atypical symptoms such as jaw and back pain, especially in males”. The accompanying text for Jane stated that ACS can present “atypically, especially in women”. The platforms' closing recommendation text also differed: John's report stated that it is crucial to seek immediate medical attention, while Jane's report stated that it is important to seek medical evaluation promptly, a less urgent formulation despite explicit acknowledgement that ACS can present without chest pain, especially in women.

On the pulmonary embolism vignette, Docus AI ranked Pneumothorax first (Moderate to High) and Pulmonary Embolism second (Moderate) for John, and ranked Pulmonary Embolism first (Moderate to High) and Pneumothorax second (Moderate) for Jane, a rank reversal favorable to Jane relative to the primary pulmonary diagnosis.

On the panic attack vignette, Docus AI ranked Panic Attack first (High) for both profiles. ACS was ranked second (Moderate) for John, and the accompanying text stated: “although you mentioned no pain in other areas, ACS can sometimes present atypically. Given your age and gender, this needs to be considered”. For Jane, ACS was ranked third (Moderate to Low), behind Cardiac Arrhythmia, and no equivalent age and gender framing sentence was generated for her.

### Ada health

On the ACS with chest pressure vignette, Ada Health ranked Heart Attack first for both profiles, with an estimated probability of 5 out of 10 for John and 4 out of 10 for Jane. Unstable Angina was ranked second for both profiles, at 8 out of 100 for John and 1 out of 10 for Jane, a higher absolute probability for the secondary diagnosis in the female profile. Both profiles' Heart Attack entries included the same text: heart attacks are slightly more common in men than women.

On the ACS without chest pressure vignette, Hyperventilation Syndrome was ranked first for both profiles and Pulmonary Embolism second. Heart Attack was ranked third for John (6 out of 100) and fifth for Jane (3 out of 100), behind Acute Respiratory Failure and Adaptive Stress Response. Jane's differential included additional sex-framing text that was not present in John's: the Heart Attack entry stated that heart attacks are slightly more common in men than women, the top-ranked Hyperventilation Syndrome entry stated that the condition tends to affect women more often than men, and the fourth-ranked Adaptive Stress Response entry stated that it is slightly more common in females than males. Both profiles' top-ranked diagnosis carried the same seek urgent medical care label; Heart Attack carried a seek emergency care label for both profiles at its respective rank position.

On the pulmonary embolism vignette, Pulmonary Embolism was ranked first for John (3 out of 10) and Heart Attack was ranked first for Jane (2 out of 10), with Pulmonary Embolism appearing third for Jane (1 out of 10).

On the panic attack vignette, both profiles received an identical top-ranked differential (Acute Panic Attack, 4 out of 10), and neither profile's top four diagnoses included Heart Attack. Ada Health posed a materially similar set of psychologically framed follow-up questions to both profiles, including inquiry regarding a recent stressful life event, fear of losing control, anxiety, and avoidance behavior. We identified one deviation from full intake equivalence: Ada Health generated a distinct, structured chest pain entry (location, tightness, radiation, intensity) for Jane's assessment, and did not generate an equivalent entry for John, who instead received only a tense chest muscles entry, despite identical vignette text being entered for both profiles. This deviation did not appear to affect the top-ranked diagnosis or its probability.

## Discussion

This structured, exploratory audit found sex-based divergence in outputs across all three AI symptom checker platforms evaluated. This divergence took several forms: differences in differential diagnosis rank, differences in assigned probability, and differences in generated explanatory or advisory text. The direction of this divergence was not uniform. Some findings were unfavorable to the female profile, such as the demotion of Heart Attack from first to third rank on Isabel's ACS with chest pressure vignette, behind a common and clinically plausible alternative diagnosis in the general population (12), and from third to fifth rank on Ada's ACS without chest pressure vignette. Other findings were favorable to the female profile, such as Isabel's reversal on the ACS without chest pressure vignette, where Heart Attack ranked first for Jane, and Docus's reversal on the pulmonary embolism vignette, where Pulmonary Embolism, rather than Pneumothorax, was ranked first for Jane. We report both directions of divergence, since a design that reported only unfavorable findings would risk overstating the uniformity of the pattern.

Our matched-profile, counterfactual-substitution design draws on methods used more broadly in natural language processing fairness research (13), including approaches such as counterfactual substitution and Winogender-style schemas (14) for detecting differential system behavior. Consistent with critiques of this literature (15), we note that our design tests only a binary male or female counterfactual and cannot detect bias mechanisms that operate through gender roles rather than through stated sex, which may independently affect a user's symptom description, word choice, or a platform's response, independent of any sex-based effect measured here.

A conceptual distinction underlies our interpretation of these findings. Substantial literature documents sex-based differences in the epidemiology of ACS presentation: women are, on average, more likely to present without chest pressure, and this population-level pattern is associated with delayed diagnosis and treatment in the clinical literature (4, 6–8). However, a person using a symptom checker to understand their own symptoms is not asking an epidemiological question. They are seeking information about themselves, and the relevant quantity for that purpose is the predictive value of a given symptom set for an individual with that presentation, not the population base rate of the presentation by sex. Evidence is mixed regarding the relative positive predictive value of chest pressure for ACS in women versus men (4, 6, 7, 16, 17); this is a genuinely unsettled empirical question, and we do not take a position on it here. Our point is narrower: whatever the true relationship is, it is a question about an individual's diagnostic likelihood, not a question that population-level prevalence data alone can answer. Where a platform's differential diagnosis rank or probability estimate for an individual symptom set changes with the patient's stated sex, and that change is not accompanied by a clinical rationale specific to the individual case, we consider this a pattern worth flagging for further investigation. We cannot confirm from the outputs alone that the platforms are misapplying population statistics rather than reflecting some other aspect of their underlying models, since we did not have access to the platforms' internal logic; this is a limitation of audit studies of this kind generally.

While it is not clear what data these AI systems relied on to reach their conclusions, it is plausible that they drew, to some extent, on the existing medical literature. Yet there are several challenges with doing so. First, there is no straightforward rubric to translate a difference by sex in the population-level prevalence of ACS into a difference in the recommendation given to a specific person; defining the level of risk that crosses the threshold for recommending urgent medical care for a possible ACS should be done on the basis of an absolute risk, not a relative risk assessment. Second, women are vastly underrepresented in clinical trials; there is much more data on men than women, and this leads to less precision regarding statements that can be made about women (18). Third, this literature is built substantially from clinical trial and cohort data describing patients who present to the emergency department or another clinical setting for care. We do not have analogous data describing the population of people who pose symptom queries to a consumer AI symptom checker, who may differ systematically from an emergency department cohort in ways that affect the predictive value of a given symptom. The predictive value of any symptom in this context is therefore considerably less certain than the clinical literature alone would suggest.

The Docus AI findings are notable for containing a mirrored, self-contradictory justification: the platform generated a sex-matched explanatory sentence for each profile, in a direction that runs counter to the clinical literature on atypical ACS presentation for the male profile, while assigning a lower probability to the female profile despite her presentation matching the pattern more classically associated with women in the cited literature (4, 6, 7). Generated natural-language justifications from large language model-based systems are not necessarily a reliable guide to the computation that produced an accompanying numeric or categorical output (19); our finding is consistent with this broader concern, though we did not have access to Docus AI's underlying architecture and cannot confirm the mechanism directly. If explanatory text can diverge from a system's own probability estimate in this way, equity audits of such systems should examine both together rather than treating plausible-sounding language as evidence that the underlying estimate is well calibrated.

We also observed a difference in urgency framing not captured by rank or probability alone. On the ACS without chest pressure vignette, Docus AI's closing recommendation described immediate medical attention as crucial for the male profile and described prompt medical evaluation as merely important for the female profile, a less urgent formulation despite Jane's presentation being the one more classically associated with atypical female ACS in the literature (4, 6, 7). Ada Health's outputs did not show an equivalent difference in urgency labeling for the top-ranked diagnosis on this vignette, since Hyperventilation Syndrome carried the same label for both profiles; Heart Attack, which carries a more urgent label, was nonetheless ranked and displayed further down the list for the female profile. How differences in urgency language or rank position do or do not translate into different real-world care-seeking behavior is a question this audit cannot answer and would require a study of user response to such outputs.

The Ada Health finding on the ACS without chest pressure vignette combines a rank demotion, a probability reduction, and sex-framing text presented directly alongside the demoted diagnosis and its top-ranked alternative. We did not test whether this juxtaposition of population-level sex framing and an individual risk estimate affects how a user interprets the output. We note it as a pattern worth testing directly in future work, since it plausibly could reinforce, rather than merely reflect, a platform's own rank and probability outputs for the person reading them.

We had initially intended to test whether Ada Health's dynamic follow-up questions differed in content by patient sex on the panic attack vignette, given that psychiatric framing embedded upstream of a differential could plausibly shape both the platform's output and the user's own interpretation of their symptoms. The two profiles' follow-up questions were materially similar, and both received the same top-ranked differential. We did identify one deviation from full equivalence: the female profile's intake generated a distinct, structured chest pain entry that the male profile's intake did not, despite identical initial symptom text. This deviation did not appear to affect the top-ranked output in this instance, but it illustrates a form of process-level variability specific to adaptive, conversational symptom checkers that static, free-text platforms like Isabel Healthcare do not exhibit, and that is harder to standardize across a matched-profile audit design.

These findings intersect with several Responsible AI principles. The rank and probability divergences raise questions of fairness and accountability in an automated system that hundreds of millions of people use as a first point of contact with the health system. The Docus AI finding raises a distinct transparency concern, since the text a user reads alongside a diagnosis may not reliably reflect the computation that produced it. The black-box nature of all three platforms limits the scope for human oversight of the specific mechanism producing these outputs, though clinician-in-the-loop review of triage recommendations, where feasible, offers a partial mitigation. We note, following related methodological discussions in the AI fairness literature (20), that observing a pattern of divergence is a necessary but not sufficient step toward addressing it, and this manuscript does not propose a specific technical remedy; we consider this an important limitation of bias-audit work in this area generally, our own included.

## Limitations

This audit has several important limitations beyond those already noted above. First, it was conducted as a single observation pass per platform and vignette; outputs from probabilistic, adaptive, and generative systems may vary across repeated sessions, and we cannot characterize how consistent the patterns reported here are over time or across sessions. Second, across all three platforms and all four vignettes, the male profile was tested immediately before the female profile, with no alternation of testing order; we cannot rule out order or session-state effects as a contributor to some of the observed divergence, particularly on the adaptive and generative platforms. Third, the vignette set is limited to four clinical scenarios constructed by the authors from the published medical literature rather than validated by an independent, blinded clinical panel; a differently constructed or independently validated vignette set might produce different results. Fourth, our design used a single male and female profile pair within a narrow age range (40–49) and a single national context (United States); we cannot speak to other age groups, geographic or health system contexts, or intersectional identities that may interact with sex to produce different patterns. Fifth, our binary male or female counterfactual design, discussed above, does not capture bias mechanisms that may operate through gender roles rather than stated sex. Sixth, all three platforms are proprietary, black-box commercial systems; their training data, underlying model architectures, and update history are not disclosed, which limits our ability to explain why any particular divergence occurred and means our candidate explanations in the Discussion remain speculative. Seventh, and importantly, we did not assess whether the differences in diagnostic rank, probability, or advisory text reported here translate into differences in real-world user behavior, care-seeking, or clinical outcomes; that translation is a necessary next step before the population-level impact of these findings can be estimated. Finally, platforms evolve rapidly, including through updates to underlying language models, and our findings reflect system behavior at the time of audit (25–26 March 2026) and may not reflect platform behavior at the time of publication.

## Conclusion

This structured, exploratory audit observed sex-based divergence in outputs across all three AI symptom checker platforms evaluated, appearing as differences in differential diagnosis rank, differences in assigned probability, and differences in generated explanatory and advisory text. This divergence was not uniformly unfavorable to the female profile; some findings favored the male profile, and some favored the female profile. The Docus AI finding of a mirrored, self-contradictory justification for opposite probability estimates given identical symptoms, and the co-occurrence of sex-framing text alongside a demoted cardiac diagnosis on Ada Health, are, in our view, the most notable and most directly testable findings. Both suggest that a system's generated explanatory text may not always reliably reflect its own underlying computation, a claim that equity audits of such systems could test directly. Given the small scale of this audit, we consider these findings hypothesis-generating rather than a demonstrated, systematic pattern of bias, and we call for larger, repeated-sampling audits, ideally supported by access to platform update logs, before firmer conclusions can be drawn. We also call for equity auditing of AI symptom checkers prior to deployment and on an ongoing basis, with attention to both the numerical outputs of these systems and the natural-language explanations and advisory text they generate.

## Data Availability

The datasets presented in this study can be found in online repositories. The names of the repository/repositories and accession number(s) can be found below: All data supporting the conclusions of this manuscript, including platform screenshots and extracted text from the audited AI symptom checker platforms (Ada Health, Isabel Healthcare, Docus AI), are publicly available in the OSF repository at https://doi.org/10.17605/OSF.IO/TRK2G. No proprietary or restricted data were used in this study.
